# Editorial: Endocrine Frailty in the Elderly

**DOI:** 10.3389/fendo.2019.00627

**Published:** 2019-09-11

**Authors:** Antonio Aversa, Fabio Monzani, Sandro La Vignera

**Affiliations:** ^1^Department of Experimental and Clinical Medicine, “Magna Graecia” University, Catanzaro, Italy; ^2^Geriatrics Unit, Department of Clinical and Experimental Medicine, University of Pisa, Pisa, Italy; ^3^Department of Clinical and Experimental Medicine, University of Catania, Catania, Italy

**Keywords:** endocrine frailty, osteoporosis, hypogonadism, diabetes, thyroid disorders

Geriatric Endocrinology represents a challenge for each of the two specialists involved. Accordingly, malpractice is common since the change of the hormone balance with aging and the presence of comorbidities. Frailty is a multifactorial clinical entity with a complex physiopathology, characterized by alterations of several functional pathways from an endocrinological point of view. The present issue of Frontiers in Endocrinology focuses on the most recent advances in the field of endocrinology of aging, particularly referring to endocrine-related frailties. The aim is to evaluate the main endocrinological changes of the elderly and their systemic clinical relapses. The structure of the special issue includes 11 reviews and two original articles, dealing with the main chapters, and hot topics of geriatric endocrinology.

Longo et al. deeply discuss the clinical management of type 2 diabetes, which represents a real challenge for the physician. They also undertake the management of glycemic goals and antihyperglycemic treatments in accordance to the medical history and comorbidities, giving preference to drugs that are associated with low risk of hypoglycemia (i.e., metformin, pioglitazone, dipeptidyl-peptidase-4 inhibitors, glucagon-like peptide 1 receptor agonists). They conclude that insulin secretagogue agents need caution because of their significant hypoglycemic risk. When used, short-acting sulfonylureas (e.g., gliclazide) or glinides (e.g., repaglinide) should be preferred.

Hypothyroidism in the elderly is another debated issue that impacts on many aspects of cognitive impairment and on surgical outcomes. Calsolaro et al. summarize the recommendations for a correct diagnostic workup and therapeutic approach to older people with increased TSH values, especially with regard to the presence of frailty, comorbidities, and poly-therapy. Vacante et al. report evidence coming from few randomized clinical trials investigating the association between non-thyroidal illness (or low-T3 syndrome) and adverse surgical outcomes. They recommend to postpone elective surgery in elderly patients with hypothyroidism until the euthyroid state is achieved. If patients need urgent or emergent surgery, it is recommended to proceed with surgery only in case of mild or moderate hypothyroidism. In this setting, a strong association between T3/T4 ratio reduction recently emerged as surrogate index of frailty and independent marker of survival (1).

Adrenal function in the elderly is a controversial issue for clinicians. In the presence of chronically increased glucocorticoid levels, normal stress response in the elderly is impaired, leading to other age-related changes, including loss of muscle mass, hypertension, osteopenia, visceral obesity, and diabetes. Yiallouris et al. discuss on the complexity of the adrenal hormone changes observed throughout the normal aging process, including surgical procedures. In contrast to the increase in glucocorticoid levels, other adrenocortical hormones, particularly serum aldosterone and DHEA, show significant decreases in the elderly. Gorini et al. provide robust evidence that aldosterone and the mineralocorticoid receptor (MR) dysregulation may play a relevant role in the control of cardiovascular and metabolic functions in the elderly by promoting vasoconstriction and acting as potent pro-fibrotic agents in cardiovascular remodeling. Also, MR contributes to increase blood pressure with aging by regulating myogenic tone, vasoconstriction, and vascular oxidative stress. In addition, dysregulation of MR signaling is associated with hypertension, obesity and diabetes, representing an important cause of increased cardiovascular risk. Plasma aldosterone concentrations decrease in the elderly as well as skeletal muscle content. Interestingly, in a human model of aldosterone excess [primary hyperaldosteronism (PA)], Kwak et al. demonstrate that skeletal muscle mass of women with PA was lower than controls, suggesting that excess of aldosterone may exert adverse effects on skeletal muscle metabolism. The clinical use of MR antagonists is limited by the adverse effects induced by MR blockade in the kidney, rising the risk of hyperkalemia in older patients with reduced renal function.

Musculoskeletal aging is a major public health concern due to high risk of falls, loss of autonomy, and institutionalization with small health outcomes. Bone mineral content is closely related with muscle mass. Several evidence suggest that osteoporosis and sarcopenia share common pathophysiological factors. Furthermore, the correlation between low bone mineral density and sarcopenia in both men and women has been showed. Accordingly, sarcopenia and osteoporosis, which are typical features of aging, are often associated with each other and with the frailty syndrome. Greco et al. investigated the interplay between frailty syndrome, typical of the older people, and the reduction in the quality of life and mobility. By contrast, Vitale et al. discussed the potential role of the IGF-1 system in the modulation of longevity, hypothesizing that the endocrine and metabolic adaptation observed in centenarians and in mammals during caloric restriction may be a physiological strategy for extending lifespan through a slower cell growth/metabolism, a better physiologic reserve capacity, a shift of cellular metabolism from cell proliferation to repair activities and a decrease in accumulation of senescent cells. In line with this clinical evidence, c-Kit, a type III tyrosine kinase receptor, is involved in multiple intracellular signaling whereby it is mainly considered a stem cell factor receptor, participating in vital functions of the mammalian body, including the human. Marino et al. found that c-kit haploinsufficiency in c-kit-deficient mice causes a worsening of myocardial repair after injury and accelerates cardiac aging, thus suggesting that the adult myocardium relies on c-kit expression to regenerate after injury and to counteract aging effects on cardiac structure and function.

Sexual and reproductive functions should be considered as complimentary issues for healthy aging. It is known that older people are interested or still engaged in sexual activities independently of gender. The age-related decline of testosterone often determines unresponsiveness to erectogenic drugs. Meta-analytic data addressed to phosphodiesterase type-5 inhibitors (PDE5i) a protective role on the cardiovascular health in patients with decreased left ventricular ejection fraction so that the addition of testosterone to a PDE5i may represent a successful strategy to prevent male sexual dysfunctions in the presence of reduced testosterone levels, as suggested by Aversa et al. By contrast, psychosocial factors play a critical role in sexual functioning of elderly women, but the anatomical and hormonal integrity of the urogenital system importantly affects many aspects of postmenopausal women's health as well, including the sexual function. A proper assessment of this system should encompass genital symptoms (dryness, burning, itching, irritation, bleeding), sexual symptoms (dyspareunia and other sexual dysfunctions) and urinary symptoms (dysuria, frequency, urgency, recurrent urinary infections). Nappi et al. recommends to fully evaluate all these aspects to enhance physical, emotional and mental well-being in elderly post-menopausal women desiring sexual life. Finally, Gallo et al. provide evidence addressing to advanced age a negative role for successful reproduction also in the male gender, by reporting that in their Assisted Reproduction Center, male age >43 years-old doubles the probability of obtaining poor quality embryos compared to younger men.

The development of neuroimaging has opened new perspectives in clinical and basic research and has modified the concept of brain aging. Tigano et al. suggest that in the near future, neuroimaging will play an increasingly important role in the definition of the individual's brain aging in every phase of the physiological and pathological process and discuss ethical and legal aspects related to precocious diagnosis of brain degenerative diseases with regard to social and clinical implications.

## Author Contributions

All authors listed have made a substantial, direct and intellectual contribution to this Editorial Article, and approved it for publication.

### Conflict of Interest Statement

The authors declare that the research was conducted in the absence of any commercial or financial relationships that could be construed as a potential conflict of interest.
